# Factors Associated with Body Size Perception and Body Image (Dis)Satisfaction in the Elderly: Results of the ELSA-Brasil Study

**DOI:** 10.3390/ijerph17186632

**Published:** 2020-09-11

**Authors:** Maria de Jesus Mendes da Fonseca, Isiyara Taverna Pimenta, Liliane da Silva Albuquerque, Estela M. L. Aquino, Letícia de Oliveira Cardoso, Dóra Chor, Rosane Harter Griep

**Affiliations:** 1 National School of Public Health, Oswaldo Cruz Foundation, Rio de Janeiro 21041-210, Brazil; isiyarataverna@gmail.com (I.T.P.); leticiadeoliveiracardoso@gmail.com (L.d.O.C.); dorachor@gmail.com (D.C.); 2 Secretary of Education, Rio de Janeiro State Government, Rio de Janeiro 20220-800, Brazil; lilianesalbuquerque@gmail.com; 3 Institute of Collective Health, Federal University of Bahia, Salvador 40110-040, Brazil; estela@ufba.br; 4 Laboratory of Health and Environment Education, Oswaldo Cruz Institute, Oswaldo Cruz Foundation, Rio de Janeiro 21040-360, Brazil; rohgriep@gmail.com

**Keywords:** body image dissatisfaction, body size perception, sociodemographic factors, behavior factors, aging process

## Abstract

The study aimed to assess the association between body image perception and (dis)satisfaction and sociodemographic and behavioral factors in the elderly, using multinomial logistic regression. Data were analyzed for 1686 women and 1499 men participating in the Brazilian Longitudinal Study of Adult Health (ELSA-Brasil). Men with less schooling and women with lower per capita income showed higher odds of underestimating their body size. Former smokers of both sexes showed higher odds of overestimating their body size; lower schooling and lower per capita income decreased these odds. Increasing age, lower per capita income, and smoking increased the odds of dissatisfaction due to thinness in men, and married marital status decreased these odds. In women, low per capita income, weekly consumption of vegetables, and smoking increased the odds of such dissatisfaction. Factors that increased the odds of dissatisfaction due to excess weight in both sexes were primary or secondary schooling and former smoking. In women, low per capita income, weak physical activity, weekly consumption of vegetables, and excessive alcohol intake also increased the odds of such dissatisfaction. The results suggest that improved living conditions and the adoption of healthy behaviors can help reduce misperceived negative body image among elderly.

## 1. Introduction

Body image, the representation of their own body that individuals form in their minds [1], consists of two components: Perceptual and attitudinal [2]. The perceptual component refers to the precision with which people judge their own appearance or physical size. Assessment of this component can identify distortions as to total size, or specific areas, of the body. Moreover, it has a behavioral component that base on behavior that is adopted because of the perception one has of his or her body image and of how we are seen by the society in which we live [2,3]. The attitudinal component involves the feelings that express whether the individual likes or dislikes his or her physical shape and appearance, from which body image (dis)satisfaction can be identified [2,3]. However, the literature places greater emphasis on negative perception or dissatisfaction with weight and appearance.

Body image is a dynamic representation that can be influenced by various factors (e.g., sociodemographic, anthropometric, behavioral, cultural, media, and health-related) [4,5,6,7,8]. Although most studies are conducted in adolescent and young adult populations [9], preoccupation with physical shape or body size is not limited to these age groups.

Among the elderly, physiological and social changes inherent to aging can affect individuals’ relationship to their bodies and lead us to expect simultaneous alterations in their body image. Physiological changes include those related to body composition and appearance, which distance the elderly individual from stereotypes of beauty and youthfulness socially idealized for both men and women [10]. The social changes involve social relations, which can be impacted by retirement, loss, and diminishing social contacts, changes in family roles, and others [11].

In this scenario, the representation of the aging body can create feelings of distress and frustration in view of losses (of beauty and health for women and of physical strength, virility, and health for men) and also of acceptance, i.e., when the individual accepts aging as a natural process [12,13]. The literature includes studies that have found the same pattern of dissatisfaction with body image in both the elderly and younger individuals, especially in women [14,15], while others have found higher satisfaction among both elderly women and men, as compared with younger people [16,17].

Although the number of studies on body image in middle-aged adults has grown in recent decades, there are still few studies in the elderly population, particularly in Brazil [18,19,20], especially studies assessing the perceptual component. A clearer understanding of the factors associated with body size perception and body image (dis)satisfaction is important for public health, given the complexity of aging and its implications for older people’s health and well-being, as well as the inconsistencies in the results of some studies, particularly among men. Our objective was thus to assess the association between body image perception and (dis)satisfaction and sociodemographic and behavioral factors in elderly men and women participating in the ELSA-Brasil study.

## 2. Materials and Methods

### 2.1. Study Design and Population

This cross-sectional study analyzed baseline data (2008–2010) from ELSA-Brasil, a multicenter cohort study consisting of 15,105 public employees ranging in age from 35 to 74 years, from six Brazilian institutions (Federal University of Bahia, Federal University of Rio Grande do Sul, University of São Paulo, Federal University of Minas Gerais, Federal University of Espírito Santo, and Oswaldo Cruz Foundation). For the baseline evaluation, participants answered a structured questionnaire addressing health and lifestyle, and socioeconomic and working conditions. Clinical and laboratory measures were also taken according to pre-established protocols. Candidates for ELSA-Brasil were excluded if they had severe communication or cognitive disorders, were currently pregnant or had been less than four months prior to recruitment or were retired and living outside the metropolitan area containing the respective study site [21,22].

In developing countries like Brazil, elderly individuals are defined as those 60 years or older [23], so the current study included elderly individuals of both sexes in the age range from 60 to 74 years (*n* = 3263) and excluded those with missing data for the target variables (*n* = 78). A total of 3185 elderly individuals were evaluated (1499 men and 1686 women).

### 2.2. Weight Status

Body weight and height were measured according to the protocol proposed by Lohman, Roche, and Martorell [24] to determine body mass index (BMI). Weight was measured with a digital scale (Toledo^®^, São Bernardo do Campo, São Paulo, Brazil) accurate to 50 g, and height (m) was measured with a fixed stadiometer (Seca^®^, Hamburg, Germany) accurate to 0.1 cm. BMI was classified as underweight (<23 kg/m^2^), normal weight (≥23 and <28 kg/m^2^), overweight (≥28 and <30 kg/m^2^), and obesity (≥30 kg/m^2^), according to criteria established for the elderly by the Pan American Health Organization for the Health, Well-Being, and Aging (SABE) study [25].

### 2.3. Body Image

Body size perception and body image (dis)satisfaction were evaluated using scales with 15 silhouettes for each sex, developed and validated for the Brazilian adult population [26,27]. The scales’ test-retest reliability was assessed in a subsample of elderly participants in the ELSA-Brasil study in the 60 to 74-year age range and was considered satisfactory [28]. The silhouettes are associated with mean BMI values ranging from 12.5 (silhouette 1) to 47.5 kg/m^2^ (silhouette 15) and include a constant BMI interval of ±1.25 kg/m^2^.

The silhouettes for each sex were numbered from 1 to 15 and shown to participants, arranged in upward order, on individual cards. Participants were asked to identify the silhouette that best represented their own body on the day of the interview (perceived silhouette) and the silhouette that represented the body they would like to have (desired silhouette).

To assess the body size perception score, the previously calculated BMI was converted into the corresponding silhouette number (actual silhouette), and the difference was calculated between the perceived silhouette and the actual silhouette. Body image (dis)satisfaction score was calculated as the difference between the perceived silhouette and the desired silhouette. Based on the scores obtained, seven categories were created for classification of these variables (Table 1).

No participant expressed serious dissatisfaction due to thinness or serious underestimation, and few expressed moderate dissatisfaction due to thinness and moderate underestimation, or serious dissatisfaction due to excess weight and serious overestimation. We thus grouped the categories for moderate and serious dissatisfaction due to excess weight, moderate and serious overestimation, moderate and mild dissatisfaction due to thinness, and moderate and mild underestimation.

The final categories of (dis)satisfaction used in the analyses were: mild and moderate dissatisfaction due to thinness, satisfaction, mild dissatisfaction due to excess weight, and moderate and serious dissatisfaction due to excess weight. The final categories for perception of body size were: Mild and moderate underestimation, no distortion, mild overestimation, and moderate and serious overestimation.

### 2.4. Sociodemographic and Behavioral Factors

Physical activity was assessed via the leisure-time and commuting domains of the long version of the International Physical Activity Questionnaire (IPAQ) [30]. According to the intensity and duration of the activities, participants were classified in three levels of physical activity: Weak (those who did no exercise at all or did not fit the other categories), moderate (those who practiced high-intensity activities three or more days a week for at least 20 min a day or moderate-intensity activities and/or walking five or more days for 30 min a day or any combination of walking and moderate or high-intensity activities, reaching a minimum of 600 MET-minute/week), and strong (high-intensity activity for three days, reaching at least 1500 MET-minutes/week, or any combination of walking and moderate or high-intensity activities reaching total physical activity of at least 3000 MET-minutes/week). MET-minute represent the amount of energy expended carrying out physical activity [31]. Since few participants reported moderate or strong physical activity, these two categories were grouped in the analyses.

Individual consumption of fruits and vegetables was assessed as a marker of quality of diet with the questions: “How often do you eat fruit?” and “How often do you eat greens or vegetables, raw, cooked, or sautéed, not including potatoes, cassava/manioc, or yams?” The answers were grouped in the following categories of consumption: High (twice or more a day), daily (once a day or five to six times a week), weekly (two to four times a week), or rarely (once a week or less) [32].

The amount of alcohol consumed per week was self-reported and categorized, by consumption in grams (g) per week, as: None, moderate (<140 g/week for women and <210 g/week for men), and excessive (≥140 g/week for women and ≥210 g/week for men) [33]. Self-reported smoking was categorized as non-smoker, former smoker, and smoker.

The independent sociodemographic variables were age (continuous variable); schooling, categorized as complete primary or less, complete secondary, and university; per capita family income (calculated by dividing total family income by the number of household members) and categorized, by tertiles, as low, middle, and high income (the income range of the sample was 1st tertile: US $678.20, 2nd tertile: US $1432.24, 3rd tertile: US $4,296,73, considering the mean exchange rate in 2009); and marital status, categorized as single, separated/divorced/widow(er), and married/living with a partner.

### 2.5. Data Analysis

The categorical variables were described as absolute and relative frequencies, and the continuous variables, as means and standard deviations. Multinomial logistic regression was used to examine the association between exposure variables (physical activity; consumption of fruits, vegetables, and alcohol; smoking; age; schooling; per capita family income; and marital status) and outcomes (body size perception and body image (dis)satisfaction). Race/skin color was also considered as an exposure variable when the outcome was body image (dis)satisfaction. Simple and multivariate analyses were performed, stratified by sex, to estimate the odds ratios and respective 95% confidence intervals (95% CI). Although the dependent variable is ordinal, in this analysis, multinomial logistic regression is the most appropriate, since the reference category is the mid-score. The multivariate models included the variables that showed association with the outcomes in the simple analyses, and the final models included the variables that remained associated in the multivariate models. The data were analyzed with the R software version 3.6.1 [34] and significance was set at 5%.

### 2.6. Ethical Aspects

The ELSA-Brasil study was approved by CONEP, the Brazilian National Research Ethics Commission (approval number 976/2006) and by the ethics committees of each institution involved. Participants who agreed to participate in the study signed the Informed Consent Form (ICF). The privacy of the data obtained was ensured.

## 3. Results

More than half of the study population (52.9%) were females. Men and women were of similar mean age (around 65 years). Although men and women of normal weight (by BMI) formed the largest category, people with excess weight (39%) came a close second. Most men and women had university schooling, weak level of physical activity, daily consumption of fruits and vegetables, and self-reported white skin color/race. There were higher proportions of married men (84.4%) and of women who did not consume alcohol (65.6%) and who had never smoked (64.8%). (Table 2).

As regards body size perception, more than half of the population (52.1%) showed mild overestimation, which was greater among women (59%). As for body image (dis)satisfaction, most participants expressed mild dissatisfaction due to excess weight (47.6%), which was also more common in women (52.2%) (Table 3).

**Figure 1:**
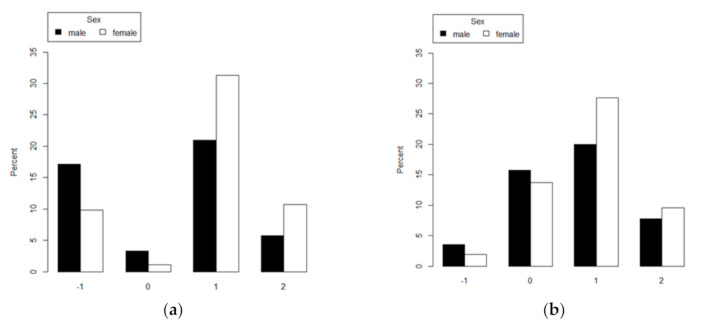
Body size perception and body image (dis)satisfaction, by sex, in elderly participants in the ELSA-Brasil study (2008–2010). (**a**) Body size perception: −1 = mild and moderate underestimation of body size; 0 = no distortion; 1 = mild overestimation of body size; 2 = moderate and serious overestimation of body size. (**b**) Body image (dis)satisfaction: −1 = mild and moderate dissatisfaction due to thinness. 0 = satisfaction. 1 = mild dissatisfaction due to excess weight. 2 = moderate and serious dissatisfaction due to excess weight.

Considering the distribution of body size perception by sex, men more often showed underestimation and no distortion than women, who mostly overestimated their body sizes. Regarding body image (dis)satisfaction, men were more satisfied and dissatisfied due to thinness than women, who were predominantly dissatisfied due to excess weight (Figure 1).

In men, considering the simple analyses and body size perception, primary schooling, low per capita income (odds ratio, OR: 2.9), and rare consumption of fruits (OR: 2.2) increased the odds of underestimating body size, while excessive alcohol consumption decreased these odds by 66%. Low schooling and low per capita income decreased the odds of mild overestimation by some 30%, while excessive alcohol consumption and former smoking increased these odds by 1.5 and 1.3 times, respectively. None of the variables was associated with moderate or serious overestimation. In the final model (Table 4), primary schooling increased threefold the odds of underestimating body size, while former smoking decreased these odds by 40%. The results showed higher odds of mild overestimation of body size among former smokers, while low schooling decreased these odds by 30%.

As regards body image (dis)satisfaction, in the simple analyses, men with primary schooling (OR: 2.3), low per capita income (OR: 2.3), and smokers (OR: 1.4) showed higher odds of dissatisfaction due to thinness. Meanwhile, these odds decreased among married individuals or those living with a partner (OR: 0.4) and those with moderate alcohol consumption (OR: 0.6). Each year of age increased the odds of dissatisfaction due to thinness by 5%. Primary schooling, secondary schooling, and low per capita income decreased by 34%, 36%, and 42%, respectively, the odds of mild dissatisfaction due to excess weight, while former smoking increased these odds by 1.4 times. Factors that increased the odds of moderate and serious dissatisfaction due to excess weight were primary or secondary schooling (OR: 2.2 and 1.8), low or middle per capita income (OR: 1.7 and 1.8), weak level of physical activity (OR: 1.6), rare consumption of fruits (OR: 1.7), weekly consumption of vegetables (OR: 1.8), and former smoking (OR: 1.8). The final association model (Table 4) showed that increasing age, low per capita income, and smoking increased the odds of dissatisfaction due to thinness, while being married or living with a partner decreased these odds by 70%. Low per capita income decreased by 41%, and former smoking increased by 49%, the odds of mild dissatisfaction due to excess weight, while weak physical activity increased the odds by 38%. The factors that increased the odds of moderate and serious dissatisfaction due to excess weight were primary or secondary schooling and former smoking.

Among women, considering the simple analyses and body size perception, the odds of underestimating body size were increased threefold by lower schooling, while middle and low per capita income increased the odds of this distortion by 4 and 4.9 times, respectively. However, primary schooling and low per capita income decreased by 30% the odds of slightly overestimating body size, while moderate alcohol consumption increased these odds by 1.4 times. The odds of moderate and serious overestimation of body size were higher among women who were former smokers (OR: 1.5) and those with excessive alcohol consumption (OR: 2.9). In the final model (Table 5), middle and low per capita income increased by 3.8 and 4.7 times, respectively, the odds of underestimating body size. Low per capita income decreased the odds of mild overestimation. Former smoking and smoking increased the odds of moderate and serious overestimation (OR: 1.5 and 1.9).

In the simple analyses of body image (dis)satisfaction, secondary schooling (OR: 2.5), low per capita income (OR: 3.2), black or mixed race/skin color (OR: 3.4 and 2.3), weak physical activity (OR: 2.3), rare consumption of fruits (OR: 3.0), weekly consumption of vegetables (OR: 5.0), and smoking (OR: 3.5) increased the odds of dissatisfaction due to thinness, while moderate alcohol consumption decreased these odds by approximately 60% in women. Women with weekly consumption of vegetables (OR: 1.5), excessive alcohol consumption (OR: 3.4), and former smokers (OR: 1.8) showed increased odds of mild dissatisfaction due to excess weight. In women, former smokers (OR: 1.8), primary or secondary schooling (OR: 2.3 and 2.6), middle and low per capita income (OR: 1.6 and 2.8), weak physical activity (OR: 2.0), rare consumption of fruits (OR: 2.9), weekly consumption of vegetables (OR: 2.5), excessive alcohol consumption (OR: 3.0), and black race/skin color (OR: 1.9) were associated with high odds of moderate and serious dissatisfaction due to excess weight, while women with moderate alcohol consumption showed a decrease of approximately 40% in these odds. In the final model (Table 5), women who defined themselves as being black, with low per capita income, weekly consumption of vegetables, and smokers showed high odds of dissatisfaction due to thinness. Moderate alcohol consumption decreased these odds by 59%. Excessive alcohol consumption and former smoking increased by 3.3 and 1.9 times, respectively, the odds of mild dissatisfaction due to excess weight. Factors that increased the odds of moderate and serious dissatisfaction due to excess weight were secondary schooling, low per capita income, weak physical activity, former smoking, weekly vegetable consumption, and excessive alcohol consumption.

## 4. Discussion

The study’s objective was to assess the association between body size perception and body image (dis)satisfaction and sociodemographic and behavioral variables in elderly men and women in the ELSA-Brasil study. In the final models, associated factors differed according to the component of body image investigated and sex. Men tended to be more satisfied and less likely to distort their body size than women. The proportion of mild or moderate/serious dissatisfaction due to excess weight was higher in women, as was the proportion of overestimation of body size (mild, moderate/serious). Meanwhile, men showed higher rates of mild/moderate dissatisfaction due to thinness and of underestimation of body size (mild/moderate) than women.

Few studies have used the 15-silhouette scales developed by Kakeshita [26] to evaluate body size perception and body image (dis)satisfaction, and none used the same categorization criterion as in the current study. However, our findings are consistent with those of previous studies. Zamacona, Poveda, and Rebato [29] assessed body image (dis)satisfaction in adult and elderly (18 to 70 years) men and women in Spain using the silhouette scales proposed by Stunkard, Sorensen, and Schulsinger [35] and also found a higher percentage of dissatisfaction due to excess weight in women than in men.

Cultural characteristics are highly important for the development of body image and favor the emergence of different perspectives for understanding and accepting the body, shape, and weight [36]. What we see in the literature is that growing industrialization, modernization, and westernization, have increasingly standardized ideals of body size among urban populations [37]. Western societies make a distinction between the idealized bodies for men and women. For men, physical strength is valued, and muscular bodies are thus considered ideal, while for women, a slim body is considered ideal [38]. In elderly individuals, the positive or negative view of the body extends beyond deeply rooted cultural values and involves issues related to functionality, health, and others, especially when the aging body is compared to younger bodies or to the recollection of one’s own body during youth [12,13]. The body image is also distorted in old age, since society has a negative view of aging, associating aging with functional disability and incompetence; this view is stronger with regard to women, who are expected to look young in order to ensure their place in society [39].

Increasing age was associated with higher odds of mild and moderate dissatisfaction due to thinness in men, which can be explained by the changes in body composition resulting from aging. The aging process involves physiological alterations that lead to decreased muscle mass and strength [40] and increased amounts of total and abdominal adipose tissue [41], so that elderly men differ from the socially idealized model of the perfect body.

Men and women with less schooling showed higher odds of moderate and serious dissatisfaction due to excess weight. These results corroborate the finding by Skopinski, Resende, and Schneider [42], in a study of middle-aged and elderly women (49 to 73 years), of a negative correlation between schooling and body image satisfaction.

Studies have found a positive correlation between BMI and body image (dis)satisfaction score and increased odds of dissatisfaction with inappropriate weight status (underweight or excess weight) in the elderly [6,42,43]. Other studies have found an inverse association between schooling and excess weight in women [44,45]. In agreement with this, we found higher prevalence of excess weight in women with lower education (54% = primary; 45.1% = secondary/values not shown), which could explain the higher odds of dissatisfaction due to excess weight in individuals with less schooling.

Women who defined themselves as being black showed increased odds of mild and moderate dissatisfaction due to thinness. Most black women in this study sample had lower income and schooling (65.4% and 85.2%, values not shown); by contrast, most white women had higher income and schooling (82.9% and 70.5%, values not shown). Inequality between blacks and whites in Brazil is related to structural factors and discrimination. When black people are in groups with less access to formal education, they also occupy less prestigious positions in the labor market [46]. Given the context of social inequalities, we suggest that black women dissatisfied due to thinness may desire larger bodies because they perceive them as synonymous with abundance and health.

Both men and women with lower income showed increased odds of mild and moderate dissatisfaction due to thinness. Low income also increased the odds of moderate and serious dissatisfaction due to excess weight in women and decreased the odds of mild dissatisfaction due to excess weight in men. Lower-income elderly may have difficulty paying their daily expenses, including those related to food [47], which increases the odds of low-quality diet [48]. This scenario can predispose the older person to lose weight (due to decreased calorie intake) or to gain weight (due to increased consumption of unhealthy and high energy-dense foods). We suggest that lower income can promote changes in weight status and consequently lead to dissatisfaction due to thinness or dissatisfaction due to excess weight, depending on the direction of weight change.

The associations between body size perception and schooling and income differed between men and women. Men with less schooling and women with lower income showed higher odds of slightly and moderately underestimating, and lower odds of slightly overestimating, body size. The results suggest that less educated elderly men and elderly women with lower income tend to perceive their bodies as smaller than they really are.

Married men or those living with a partner showed lower odds of mild and moderate dissatisfaction due to thinness. The definitions of gender roles in housework have changed over the years, but despite the increase in men’s participation, women are still in charge of most of the household chores and care for the children [49]. The wives’ contribution to the husbands’ diet may have been a factor in the lower rate of underweight in married men or those with partners, compared to single men (14.8% and 28.3% respectively, for married/with partner versus single, results not shown) and can help explain the observed results. As in the current study, Kops, Bessel, Knauth, Caleffi, and Wendland [50] did not observe an association between marital status and body image dissatisfaction in Brazilian adult women and elderly women (25 to 82 years).

Men and women with low levels of physical activity showed increased odds of mild dissatisfaction and moderate and serious dissatisfaction due to excess weight, respectively. Physical activity provides numerous benefits in the elderly, including prevention and treatment of diseases and disabilities [51]. A study of elderly Brazilian women (60 years or older) showed that those practicing regular resistance training expressed greater satisfaction with their body image and greater functional strength in the upper and lower limbs, compared to inactive women [52]. Most elderly men and women showed low levels of physical activity (a category that included individuals with no physical activity whatsoever), and among them there were higher rates of overweight and obesity (results not shown). This raises concerns about the health and well-being of participants in the ELSA-Brasil study and emphasizes the need for strategies to promote physical activity.

Weekly consumption of vegetables increased the odds of mild and moderate dissatisfaction due to thinness and moderate and serious dissatisfaction due to excess weight in women. Decreased consumption of vegetables may be associated with lower energy intake and could lead to underweight, explaining the increased odds of dissatisfaction due to thinness. However, individuals with lower consumption of vegetables may present increased energy intake due to the substitution of these healthy foods with unhealthy or ultra-processed products, the consumption of which has been associated directly with excess weight [53]. This suggests that the consumption of unhealthy and higher energy-dense foods has led to weight gain and increased odds of dissatisfaction with the observed excess weight. However, it is possible that women who are dissatisfied with excess weight are more likely to adopt unhealthy eating behaviors as the negative result of body image dissatisfaction, as observed by Oliveira et al. [8] in adult and elderly Brazilian women (33 to 79 years).

Moderate alcohol consumption decreased the odds of mild and moderate dissatisfaction due to thinness, while excessive consumption increased the odds of mild, moderate, and serious dissatisfaction due to excess weight in women. The increase in energy intake through alcohol consumption can favor weight gain [54] and explain the higher odds of dissatisfaction due to excess weight. Although men showed more frequent consumption of alcoholic beverages, no associations were found in these individuals. Albuquerque [7] found different results: Excessive alcohol consumption was associated with 1.9 times greater odds of dissatisfaction due to excess weight and 1.5 greater odds of dissatisfaction due to thinness among men from 35 to 59 years of age participating in the ELSA-Brasil study.

Former smokers showed higher odds of mild, moderate, and serious dissatisfaction due to excess weight in both sexes, mild and moderate underestimation and mild body size overestimation in men, and moderate and serious body size overestimation in women. Smoking increased the odds of mild and moderate dissatisfaction due to thinness in both sexes and moderate and serious overestimation among women. Nicotine increases the body’s energy expenditure and can decrease appetite, favoring weight loss in smokers. Meanwhile, smoking cessation can contribute to weight gain due to decreased energy expenditure and increased energy intake [55]. The suggestion is that weight gain and thus increasing body size in former smokers could lead to dissatisfaction due to excess weight and overestimation of body size. But, if weight gain happened quickly, elderly men may not have noticed this change in body size and would, therefore, underestimate their body sizes. Meanwhile, the weight loss that can be observed in smokers could explain the dissatisfaction due to thinness in men and women; depending on the speed of weight loss, elderly women may have difficulty correctly perceiving their body size and overestimate it. Higher odds of dissatisfaction due to excess weight in former smokers were also observed in Brazilian adults of both sexes [7].

The current study has some limitations. The use of two questions as markers of quality of diet may have overestimated or underestimated the frequency of consumption of fruits and vegetables. The shortage of studies on body image using the silhouette scales developed by Kakeshita [27] and focusing exclusively on the elderly population hindered discussion of the results, while highlighting the current study’s unique approach, in that it evaluated an elderly population. This evaluation was limited at baseline, because the scale used was applied only at this later stage of the study. Moreover, the cross-sectional design of the study does not allow causality to be inferred, and the use self-reported variables is a possible source of imprecision.

Despite these limitations, this is the first study to propose categories considering degrees of body image dissatisfaction and distortion based on the use of the Kakeshita silhouette scales [27]. Given the shortage of studies on body image and especially ones evaluating the perceptual component in the elderly population, this article helps fill important knowledge gaps. The reliability of the data collected is also guaranteed by the quality control of the ELSA-Brasil study before, during, and after the data collection, through training of the research team, development of an operations manual, pretesting of the instruments, and other procedures [21].

## 5. Conclusions

In summary, different factors were associated with body size perception and body image (dis)satisfaction in elderly men and women in the ELSA-Brasil study. In men, the variables associated with body size perception were schooling and smoking and the variables associated with (dis)satisfaction were age, schooling, per capita income, marital status, physical activity, and former smoking. In women, the factors associated with body size perception were per capita income and smoking, and those associated with (dis)satisfaction were schooling, per capita income, race/skin color, physical activity, consumption of vegetables, alcohol consumption, and former and current smoking. The patterns of (dis)satisfaction in both sexes was similar to findings in younger adults.

Positive body image, whatever the component evaluated, is important for decreasing risky behaviors aimed at achieving the desired body, as in eating disorders, restrictive and nutritionally imbalanced diets, and other behaviors that, although more common among younger people, are also observed, to a lesser extent, among the elderly; the ideal of beauty and youth valued by younger people persists even with advancing age, creating a negative view of aging associated with loss of beauty, health, and physical fitness. In this context, it is important to redefine old age from a positive perspective, since it is a stage of life when maturity is achieved, in which it is possible to share living experiences acquired over the years and elaborate new purposes in life, to re-begin if necessary. Acceptance of the body changes that occur over the years and an ideal of beauty that is disassociated from what is recommended for younger people can improve negative images held in this age group [39].

This paper contributes to understanding factors that are associated with dissatisfaction and perception of body image in the elderly. Most of the socioeconomic and behavioral characteristics that were associated with body image in this study are changeable. Public policies that promote improvements in living conditions and the adoption of healthy behaviors can help mitigate body image dissatisfaction and distortion in the elderly population.

## Figures and Tables

**Table 1:** Categories of body size perception and body image (dis)satisfaction.

Score	Perception (Perceived Silhouette—Actual Silhouette)	(Dis)Satisfaction (Perceived Silhouette—Desired Silhouette)
≤−8	Serious underestimation	Serious dissatisfaction due to thinness
≥−7 and ≤−5	Moderate underestimation	Moderate dissatisfaction due to thinness
≥−4 and ≤−2	Mild underestimation	Mild dissatisfaction due to thinness
≥−1 and ≤1	No distortion	Satisfaction
≥2 and ≤4	Mild overestimation	Mild dissatisfaction due to excess weight
≥5 and ≤7	Moderate overestimation	Moderate dissatisfaction due to excess weight
≥8	Serious overestimation	Serious dissatisfaction due to excess weight

Source: Adapted from Zamacona et al. [29] and Oliveira et al. [8].

**Table 2:** Sociodemographic, anthropometric, and behavioral characteristics of elderly participants in the Brazilian Longitudinal Study of Adult Health (ELSA-Brasil) study (2008–2010).

Variables	Men		Women		Total	
	*n*	%	*n*	%	*n*	%
**Population**	1499	47.06	1686	52.94	3185	100
**Age (mean ± SD)**	65.57 ± 4.31		64.83 ± 3.90		65.17 ± 4.11	
**BMI**						
Underweight	231	15.41	287	17.02	518	16.26
Normal weight	735	49.03	702	41.64	1437	45.12
Overweight	241	16.08	233	13.82	474	14.88
Obesity	292	19.48	464	27.52	756	23.74
**Schooling**						
University	893	59.57	868	51.48	1761	55.29
Secondary	269	17.95	492	29.18	761	23.89
Primary	337	22.48	326	19.34	663	20.82
**Per capita income**						
High	398	26.55	552	32.74	950	29.83
Middle	608	40.56	545	32.33	1153	36.20
Low	493	32.89	589	34.93	1082	33.97
**Marital status**						
Single	60	4.00	261	15.48	321	10.08
Separated/Divorced/Widow(er)	174	11.61	713	42.29	887	27.85
Married/With Partner	1265	84.39	712	42.23	1977	62.07
**Race/skin color**						
White	911	64.70	904	56.92	1815	60.58
Black	181	12.80	283	17.82	464	15.48
Mixed race	316	22.44	401	25.25	707	23.93
**Physical activity**						
Heavy and moderate	480	32.02	401	23.78	881	27.66
Weak	1019	67.98	1285	76.22	2304	72.34
**Consumption of fruits**						
High	350	23.35	632	37.49	982	30.83
Daily	692	46.16	774	45.91	1466	46.03
Weekly	276	18.41	179	10.62	455	14.29
Rare	181	12.07	101	5.99	282	8.85
**Consumption of vegetables**						
High	188	12.54	305	18.09	493	15.48
Daily	734	48.97	906	53.74	1640	51.49
Weekly	349	23.28	328	19.45	677	21.26
Rare	228	15.21	147	8.72	375	11.77
**Alcohol consumption**						
None	543	36.22	1106	65.60	1649	51.77
Moderate	775	51.70	525	31.14	1300	40.82
Excessive	181	12.07	55	3.26	236	7.41
**Smoking**						
Never smoked	585	39.03	1093	64.83	1678	52.68
Former smoker	748	49.90	461	27.34	1209	37.96
Smoker	166	11.07	132	7.83	298	9.36

SD = standard deviation, BMI = body mass index.

**Table 3:** Frequency of body size perceptions and body image (dis)satisfaction, by sex, in elderly participants in the ELSA-Brasil study (2008–2010).

Variables	Men		Women		Total	
	*n*	%	*n*	%	*n*	%
**Population**	1499	47.06	1686	52.94	3185	100
**Perception**						
No distortion	544	36.29	312	18.51	856	26.88
Mild and moderate underestimation of body size	105	7.00	35	2.08	140	4.40
Mild overestimation of body size	666	44.43	995	59.02	1661	52.15
Moderate and serious overestimation of body size	183	12.21	340	20.17	523	16.42
NA ^1^	1	0.07	4	0.24	5	0.16
**(Dis)satisfaction**						
Satisfaction	502	33.49	438	25.98	940	29.51
Mild and moderate dissatisfaction due to thinness	112	7.47	62	3.68	174	5.46
Mild dissatisfaction due to excess weight	636	42.43	880	52.19	1516	47.60
Moderate and serious dissatisfaction due to excess weight	249	16.61	306	18.15	555	17.43

NA = not applicable. ^1^ Participants with BMI outside upper limit of the silhouette scales.

**Table 4:** Final model for association between body size perception and body image (dis)satisfaction and sociodemographic and behavior factors in elderly men participating in the ELSA-Brasil study (2008–2010).

Men	Body Size Perception			Body Image (Dis)Satisfaction		
	Mild and Moderate Underestimation	Mild Overestimation	Moderate and Serious Overestimation	Mild and Moderate Dissatisfaction due to Thinness	Mild Dissatisfaction due to Excess Weight	Moderate and Severe Dissatisfaction due to Excess Weight
Variables	Adjusted OR (95% CI)	Adjusted OR (95% CI)	Adjusted OR (95% CI)	Adjusted OR (95% CI)	Adjusted OR (95% CI)	Adjusted OR (95% CI)
**Age**				**1.07 (1.02–1.13)**	1.0 (0.97–1.02)	0.98 (0.94–1.02)
**Schooling**						
University	1	1	1	1	1	1
Secondary	1.70 (0.95–3.04)	0.79 (0.58–1.08)	1.08 (0.69–1.68)	1.18 (0.62–2.26)	0.83 (0.57–1.21)	**2.06 (1.31–3.24)**
Primary	**3.12 (1.92–5.07)**	**0.72 (0.54–0.96)**	1.05 (0.69–1.59)	1.59 (0.83–3.03)	0.85 (0.57–1.28)	**2.60 (1.61–4.20)**
**Per capita income**						
High				1	1	1
Middle				1.56 (0.83–2.94)	1.02 (0.76–1.38)	1.35 (0.87–2.09)
Low				**2.19 (1.03–4.64)**	**0.59 (0.39–0.89)**	0.78 (0.45–1.34)
**Marital status**						
Single				1	1	1
Married/with partner				**0.30 (0.12–0.71)**	0.91 (0.48–1.72)	0.94 (0.39–2.26)
Separated/divorced/widower				0.52 (0.19–1.44)	1.18 (0.58–2.40)	1.24 (0.47–3.28)
**Physical activity**						
Strong and moderate				1	1	1
Weak				1.07 (0.67–1.7)	**1.38 (1.07–1.79)**	1.42 (1.0–2.01)
**Smoking**						
Never smoked	1	1	1	1	1	1
Former smoker	**0.62 (0.39–0.99)**	**1.33 (1.04–1.69)**	1.41 (0.98–2.03)	0.74 (0.46–1.20)	**1.49 (1.16–1.92)**	**1.61 (1.15–2.26)**
Smoker	0.99 (0.53–1.84)	0.88 (0.59–1.31)	1.05 (0.59–1.87)	**1.87 (1.06–3.30)**	0.77 (0.50–1.17)	0.88 (0.51–1.51)

OR = odds ratio. 95% CI = 95% confidence interval. The bold values indicate that there was statistical significance.

**Table 5:** Final model for association between body size perception and body image (dis)satisfaction and sociodemographic and behavior factors in elderly women participating in the ELSA-Brasil study (2008–2010).

Women	Body Size Perception			Body Image (Dis)Satisfaction		
	Mild and Moderate Underestimation	Mild Overestimation	Moderate and Serious Overestimation	Mild and Moderate Dissatisfaction due to Thinness	Mild Dissatisfaction due to Excess Weight	Moderate and Serious Dissatisfaction due to Excess Weight
Variables	Adjusted OR (95% CI)	Adjusted OR (95% CI)	Adjusted OR (95% CI)	Adjusted OR (95% CI)	Adjusted OR (95% CI)	Adjusted OR (95% CI)
**Schooling**						
University				1	1	1
Secondary				1.01(0.48–2.12)	0.90 (0.60–1.25)	**1.59 (1.05–2.41)**
Primary				0.54 (0.21–1.36)	0.83 (0.55,1.24)	1.49 (0.90–2.44)
**Per capita income**						
High	1	1	1	1	1	1
Middle	**3.85 (1.05–14.1)**	0.92 (0.67–1.28)	1.26 (0.85–1.87)	1.32 (0.57–3.04)	1.08 (0.81–1.46)	1.41 (0.94–2.13)
Low	**4.72 (1.36–16.37)**	**0.69 (0.51–0.94)**	0.88 (0.60–1.29)	**2.01 (0.82–4.92)**	1.47 (1.0-,2.14)	**1.77 (1.10–2.85)**
**Race/skin color**						
White				1	1	1
Black				**2.62 (1.17–5.87)**	0.70 (0.48–1.03)	1.13 (0.72–1.77)
Mixed race				1.65 (0.78–3.52)	0.79 (0.57–1.08)	0.89 (0.60–1.33)
**Physical activity**						
Strong and moderate				1	1	1
Weak				2.01 (0.86–4.68)	1.12 (0.85–1.47)	**1.73 (1.16–2.58)**
**Consumption of vegetables**						
High				1	1	1
Daily				2.45 (0.82–7.34)	1.09 (0.79–1.49)	1.26 (0.81–1.94)
Weekly				**5.19(1.65–16.28)**	1.53 (1.01–2.31)	**1.89 (1.12–3.19)**
Rare				2.18 (0.56–8.49)	1.03 (0.62–1.7)	1.21 (0.64–2.27)
**Alcohol consumption**						
None				1	1	1
Moderate				**0.41 (0.19–0.89)**	0.84 (0.64–1.09)	**0.67 (0.47–0.96)**
Excessive				3.52(0.68–18.36)	**3.30 (1.14–9.55)**	**4.59(1.42–14.86)**
**Smoking**						
Never smoked	1	1	1	1	1	1
Former smoker	0.46 (0.16–1.37)	1.28 (0.95–1.73)	**1.50 (1.05–2.14)**	1.09 (0.50–2.39)	**1.91 (1.42–2.56)**	**2.09 (1.45–3.01)**
Smoker	1.35(0.37–4.90)	1.54 (0.90–2.63)	**1.88 (1.02–3.46)**	**4.90(2.12–11.36)**	1.53 (0.93–2.53)	1.34 (0.70–2.55)

OR = odds ratio. 95% CI = 95% confidence interval. The bold values indicate that there was statistical significance.
